# Effect of non-enzymatic glycation on collagen nanoscale mechanisms in diabetic and age-related bone fragility

**DOI:** 10.32604/biocell.2023.028014

**Published:** 2023-06-21

**Authors:** James L. ROSENBERG, William WOOLLEY, Ihsan ELNUNU, Julia KAMML, David S. KAMMER, Claire ACEVEDO

**Affiliations:** 1 Department of Mechanical Engineering, University of Utah, Salt Lake City, 84112, USA; 2 Institute for Building Materials, ETH Zurich, Zurich, Switzerland; 3 Department of Biomedical Engineering, University of Utah, Salt Lake City, 84112, USA

**Keywords:** Bone fragility, Advanced glycation end-products, Collagen, Diabetes, Aging

## Abstract

Age and diabetes have long been known to induce an oxidative reaction between glucose and collagen, leading to the accumulation of advanced glycation end-products (AGEs) cross-links in collagenous tissues. More recently, AGEs content has been related to loss of bone quality, independent of bone mass, and increased fracture risk with aging and diabetes. Loss of bone quality is mostly attributed to changes in material properties, structural organization, or cellular remodeling. Though all these factors play a role in bone fragility disease, some common recurring patterns can be found between diabetic and age-related bone fragility. The main pattern we will discuss in this viewpoint is the increase of fibrillar collagen stiffness and loss of collagen-induced plasticity with AGE accumulation. This study focused on recent related experimental studies and discusses the correlation between fluorescent AGEs content at the molecular and fibrillar scales, collagen deformation mechanisms at the nanoscale, and resistance to bone fracture at the macroscale.

## Introduction

Age and type 2 diabetes mellitus (T2DM) are both associated with a drastic increase in fracture risk, in the range of 20% to 900%, independent of bone mass (Hui *et al*., 1988; Marshall *et al*., 1996; Bonds *et al*., 2006; Janghorbani *et al*., 2007; Vestergaard, 2007; Karim and Bouxsein, 2016; Liang and Chikritzhs, 2016). T2DM disproportionately affects the elderly population (those over 65 years) compared to other adults worldwide, and can lead to increased fragility of bones in older individuals (on U.S. Department of Health and Human Services, 2012; Kirkman *et al*., 2012; Bradley and Hsueh, 2016; Looker *et al*., 2016). With the global epidemic of diabetes and the aging of the population, understanding the mechanisms underlying diabetic and age-related bone fragility is urgent.

Since the early 1970s, collagen structural degradation and collagen cross-linking with age and diabetes have been amply documented in different tissues such as skin, joints, arteries, bone, or tendons (Hamlin *et al*., 1975; Piez, 1968; Traub and Piez, 1971; Robins and Bailey, 1972; Cannon and Davison, 1973; Barnes *et al*., 1974; Kivirikko, 1974; Tanzer, 1976; Cannon and Davison, 1977; Cerami *et al*., 1979; Madia *et al*., 1979). These studies have presented robust evidence that collagen (the most abundant protein in the human body) is modified by non-enzymatic glycation (Maillard reaction) between glucose and proteins and results in advanced glycation end-products (AGEs) cross-links formation. Non-enzymatic AGE cross-links within and in-between collagen molecules are often measured as bulk and comprised of pentosidine, carboxymethyllysine (CML), carboxyethyllysine (CEL), crossline, and vesperlysines (A, B, and C). Due to the long half-life of collagen, AGEs can accumulate with age. This process is quite similar but accelerated in T2DM, explaining why diabetes is often presented as a “fast aging” process (Hamlin *et al*., 1975; Monnier and Cerami, 1981; Monnier *et al*., 1984). Thirty years later, studies on bone fragility started to emerge and implicate the accumulation of AGEs in the pathogenesis of diabetes and loss of bone quality due to aging (Catanese *et al*., 1999; Vashishth *et al*., 2001; Tang *et al*., 2007; Vashishth, 2007; Saito and Marumo, 2010; Tang and Vashishth, 2011; Yamagishi, 2011; Zimmermann *et al*., 2011; Garnero, 2012). This new discovery shifted the research on bone fragility by suggesting new factors affecting bone quality at the collagen level, and new potential therapeutic targets to inhibit AGEs formation (such as blocking the receptor for AGEs [RAGE], for instance). Indeed, loss of bone mass and mineral content could not fully explain osteoporotic fracture with age (Schuit *et al*., 2004) or address diabetic bone fragility where bone mass is mostly unchanged. In this viewpoint, we discuss here how AGEs accumulation affects bone resistance to fracture (i.e., toughness) and disrupt nanoscale mechanisms of collagen deformation and energy dissipation.

## Multiscale Origins of Bone Fragility

**FIGURE 1.**
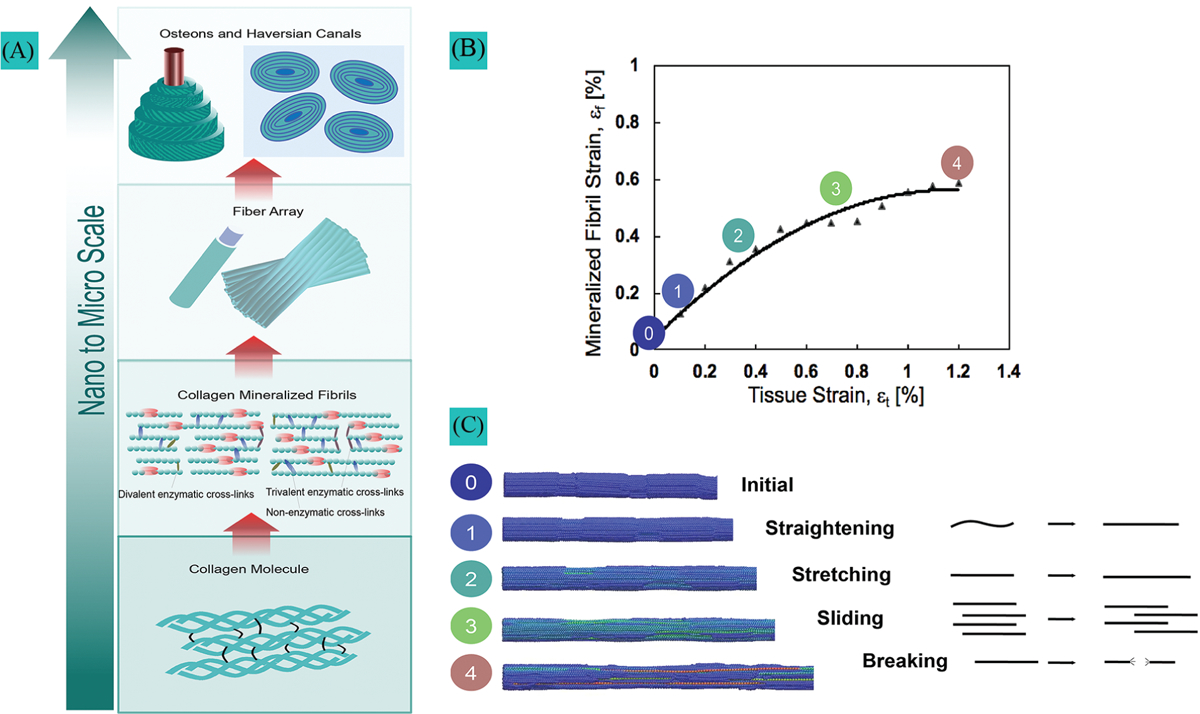
Hierarchical organization in bone and mechanisms of collagen fibril deformation. (A) Hierarchical bone structure with cross-links in the molecular and fibrillar structure of collagen. (B) Collagen fibril deformation measured by small-angle X-ray scattering during rat ulna tensile testing. (C) Collagen loading stages from the initial state to complete failure.

The ability of cortical bone to resist fracture originates from its multiscale hierarchical organization (Fig. 1A). Building blocks in bone are organized from the molecular and nanoscopic levels to the macroscopic level. At the nanoscale level, basic building blocks are formed by mineralized collagen fibrils comprising collagen molecules, hydroxyapatite crystals, and water. Mineralized fibrils are aligned, stacked in fibers, and arranged in lamellae, where each lamella layer has a different fiber orientation than its neighbor. At the microscale, concentric lamellae form tube-like structures called osteons, which protect central canals where blood vessels and nerves are running (Fig. 1A). Bone quality, used to characterize bone resistance independently of bone mass, is related to (1) material properties, (2) structure, and (3) remodeling. These three factors of bone quality are impaired with age and T2DM.

### Bone material properties

These properties (e.g., tissue modulus, yield stress/strain, ultimate stress/strain, strain to failure, work-to-fracture, and toughness) are often measured using strength and toughness tests. Strength tests are designed to measure the maximum load a material can withstand before breaking or deforming permanently. Toughness tests, on the other hand, measure the ability of a material to absorb energy before breaking (Ritchie, 2011). Material properties are conferred at the nanoscale by collagen fibrils (100 nm diameter) reinforced by mineral crystals; elastic and pre-yield properties are attributed to the stiffness of the mineral, whereas the plastic, post-yield properties, and intrinsic toughness are attributed to the ability of collagen to deform plastically and prevent crack initiation and growth (Burstein *et al*., 1975; Nalla *et al*., 2004; Currey *et al*., 2007; Launey *et al*., 2010). Most studies have shown that aging does not significantly impair the elastic modulus of bone (Nyman *et al*., 2007; Koester *et al*., 2011; Zimmermann *et al*., 2011). However, yield strength and ultimate strength are decreased, respectively, by approximately 1% and 2% per decade in human cortical bone for adults older than 30 years of age (Zimmermann *et al*., 2011; Morgan *et al*., 2018). Even more significantly, toughness, energy dissipation, and ultimate strain are reduced by approximately 10%–15% every decade (Burstein *et al*., 1976; Nalla *et al*., 2004; Nyman *et al*., 2007; Koester *et al*., 2011; Zimmermann *et al*., 2011; Morgan *et al*., 2018). Studies on material properties in diabetic human bones are not as abundant as those in aging bones, but animal studies show evidence of a significant decrease in post-yield properties, including a reduction of 10%–15% in ultimate strength and 30%–40% in ultimate strain and toughness (Campbell *et al*., 2016; Acevedo *et al*., 2018a). Based on comparative models (Nyman *et al*., 2007; Acevedo *et al*., 2018a), the reduced post-yield properties and toughness in aging and diabetic bones seem to be mostly attributable to deficits in material properties (conferred at the collagen level) rather than structural deficits.

### Bone structure

The structure of bones (microarchitecture and geometry) is also known to influence resistance to fracture and energy dissipation during crack growth (i.e., extrinsic toughening mechanisms). Extrinsic toughening mechanisms act at the microscale level to reduce the driving force that propagates the crack. These mechanisms include crack deflection/ twisting and crack bridging (Ager *et al*., 2006; Peterlik *et al*., 2006; Ritchie *et al*., 2006). When the crack propagates transversely to the osteons, crack deflection occurs around the hypermineralized osteonal boundaries (i.e., cement lines) to dissipate energy (Martin and Burr, 1982; Nalla *et al*., 2005). Other microstructural features, such as osteocyte lacunae or porosity, can deflect the crack (Currey, 1962). When the crack propagates longitudinally along the osteons (in-between lamellae and fibers), crack bridging leaves uncracked regions to resist fracture along the crack path. Aged bone shows a significant increase in osteon density by 100% to 200% between 30 to 80 years of age and an increase in vascular canal diameters (Busse *et al*., 2010; Zimmermann *et al*., 2011; Jast and Jasiuk, 2013), which vastly expand the area of cement lines at which microcracks can form, resulting in both smaller crack bridges and reduced crack deflection (Koester *et al*., 2011; Zimmermann *et al*., 2011). Diabetes moderately affects cortical bone geometry, such as a change in moment of inertia due to a different distribution of bone mass (Prisby *et al*., 2008; Acevedo *et al*., 2018a). Recent studies (Ay *et al*., 2020) have found that hyperglycemia increases osteocyte lacunar density and decreases vascular canal diameter. Changes in microstructure are closely tied to cellular remodeling, as the process of cellular remodeling involves the removal and formation of bone tissue, which can alter bone microstructure.

### Cellular-regulated remodeling

Bone health is maintained through dynamic remodeling executed by resident cells in this tissue: osteoclasts resorb old bone, followed by osteoblast deposition of bone. The osteocyte, a third bone cell type, directs osteoclast and osteoblast activity and dynamically remodels their local bone matrix in a process called perilacunar remodeling (Qing *et al*., 2012; Tang *et al*., 2012). Disruption of remodeling causes an increased risk of fracture due to bone fragility, even with normal bone mass. Age is known to create an imbalance between osteoclasts and osteoblast activity, leading to bone loss, as well as elevated osteoblasts and osteocyte apoptosis. T2DM reduces osteoblast differentiation and osteoblast-induced bone formation while enhancing osteoclast activity and increased bone resorption, which can also lead to bone loss (Lu *et al*., 2003; Suzuki *et al*., 2005; Alikhani *et al*., 2007; Liu *et al*., 2006; Takizawa *et al*., 2008). It is also thought that hyperglycemia in T2DM might induce osteocyte apoptosis (Picke *et al*., 2019). Disruption of osteocyte-mediated remodeling causes distinctive collagen disorganization and a pattern of hypermineralization of the mid-cortical bone matrix (Qing *et al*., 2012; Tang *et al*., 2012). In the following discussion, we further explore how the three factors of bone quality are affected by the increase of AGEs with age and diabetes.

## Accumulation of Advance Glycation End-Products in Collagen

To understand how AGEs accumulate in collagen, we must first understand how collagen is structured. Collagen is composed of three chains with a repeating sequence of gly-X-Y, where X/Y can be any amino acid but is generally proline or hydroxyproline. During bone development and maturation (before the age of 20 years), enzymatic cross-links are created to stabilize the triple-helix of collagen molecules and connect collagen fibrils. Enzymes, such as lysyl oxidase, prolyl hydroxylase, galactosyltransferase, and matrix metalloproteinase, play a crucial role in regulating the formation and degradation of enzymatic cross-links in bone, which is vital for maintaining optimal bone strength and remodeling. These enzymes work together to balance the formation and breakdown of cross-links, providing stiffness and strength to the bone matrix (Knott and Bailey, 1998). Enzymatic cross-links are first divalent (immature) and then trivalent (mature) cross-links that link collagen molecules head to tail. Increased enzymatic cross-link density and maturity can beneficially increase collagen fibril stiffness and bone stiffness and strength (Oxlund *et al*., 1995; Bailey *et al*., 1998; Depalle *et al*., 2015).

After bone maturation, non-enzymatic cross-links are created by an oxidative reaction between glucose and amino acid residues on proteins (e.g., lysine or hydroxylysine in type I collagen). This reaction, called the Maillard reaction (Sroga *et al*., 2015), forms cross-links among AGEs along the triple helical region of the molecule. Since AGEs are not regulated by enzymes, they can accumulate (Valcourt *et al*., 2007), especially in aging tissues with low turnover or in the presence of high blood sugar concentrations (like in T2DM) or oxidative stress. The content of AGEs, specifically the content of the well-studied pentosidine, increases with age and T2DM in cortical and trabecular bone (Tang *et al*., 2007; Karim *et al*., 2013). Contrary to enzymatic cross-links, the accumulation of AGEs with aging or diabetic cortical bone is associated with increased mechanical fragility, as will be discussed in the following section (Vashishth *et al*., 2001; Wang *et al*., 2002; Garnero *et al*., 2006; Siegmund *et al*., 2008; Silva *et al*., 2009; Saito and Marumo, 2010; Tang and Vashishth, 2010; Ionova-Martin *et al*., 2011; Snedeker and Gautieri, 2014; Acevedo *et al*., 2018a).

## Advance Glycation End-Products Correlate with Loss of Collagen Deformation and Loss of Bone Toughness

The best explanation for the decrease in plastic strain energy in bone fragility is the impairment of collagen deformation by AGEs accumulation (Nyman *et al*., 2007; Acevedo *et al*., 2018a). The current state-of-the-art technique for quantifying collagen deformation at the nanoscale is *in situ* small-angle x-ray scattering (SAXS). The scattering pattern of the collagen fibrils (d-spacing) can be used to measure fibrillar strain, while digital image correlation of bone samples can be used to measure the tissue strain at the macroscale. Only a few studies (Barth *et al*., 2011; Zimmermann *et al*., 2011, 2016; Acevedo *et al*., 2015, 2018a) have gathered data on bone fluorescent AGEs concentration at the molecular and fibrillar levels, on collagen deformation mechanisms at the nanoscale (using SAXS), and bone fracture resistance at the macroscale. These studies investigated the effect of age, diabetes, long-term alendronate treatment, and irradiation, which can affect the collagen structure and increase AGEs content.

A novel finding of these studies was to identify the different stages of fibril deformation in cortical bone and how aging and T2DM affect these mechanisms. The results indicated that factors such as age, diabetes, band high AGEs content did not influence the fibrillar straightening and stretching (stages 1 and 2, Figs. 1B and 1C) that occurs at lower levels of tissue strain, which typically precedes tissue yielding. However, the process of fibrillar sliding (stage 3, Figs. 1B and 1C), which occurs when fibrils decouple during tissue yielding, was systematically impaired with high AGEs content in these studies (Zimmermann *et al*., 2011; Acevedo *et al*., 2015, 2018a). Fibrillar sliding was found to be essential to promote plasticity at the nanoscale as the main intrinsic toughening mechanism to resist crack initiation and growth. SAXS results suggest that age (Zimmermann *et al*., 2011; Barth *et al*., 2011; Zimmermann *et al*., 2016), diabetes (Acevedo *et al*., 2018a), and exposure to x-ray radiation (Barth *et al*., 2011) limit the ability of collagen fibrils to deform plastically by fibrillar sliding due to the accumulation of AGEs. Aging and diabetes stiffen the fibrils and inhibit fibrillar sliding (Zimmermann *et al*., 2011; Acevedo *et al*., 2018a, 2018b). This phenomenon, called collagen stiffening, prevents the collagen in aged- and T2DM-affected bone from sustaining as much tissue deformation without brittle failure. The deficit in collagen ductility must be compensated at higher length scales (micro-scale) via energy dissipation through extrinsic toughening mechanisms such as crack deflection and microdamage accumulation. Evidence revealed that age, diabetes, and the AGEs content correlate with greater microdamage and microcrack accumulation (Schaffler *et al*., 1995; Diab *et al*., 2005, 2006; Diab and Vashishth, 2007; Mohsin *et al*., 2019; Liu *et al*., 2020).

**FIGURE 2.**
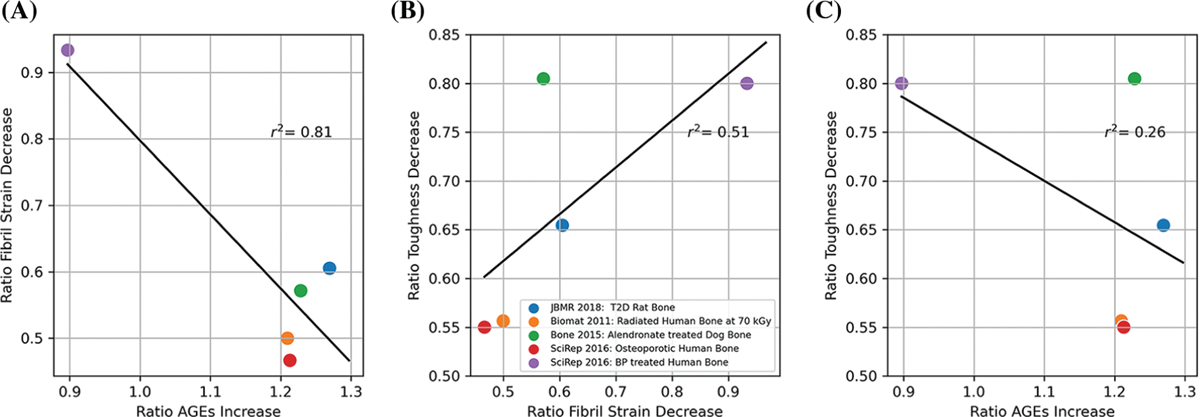
(A) Correlation between change in ultimate fibril strain and advance glycation end-product (AGE) accumulation. (B) Correlation between change in toughness and change in fibril strain. (C) Correlation between the decrease in toughness and AGE accumulation. The ratios were obtained from published values for age (Zimmermann *et al*., 2011, 2016), diabetes (Acevedo *et al*., 2018a), bisphosphonate treatment (Acevedo *et al*., 2015; Zimmermann *et al*., 2016), and irradiation (Barth *et al*., 2011).

Cumulatively, these studies show that the increase of AGEs (compared with healthy controls (Barth *et al*., 2011; Acevedo *et al*., 2015; Zimmermann *et al*., 2016; Acevedo *et al*., 2018a)) or young (Zimmermann *et al*., 2011) is highly correlated with fibrillar stiffening (r^2^ = 0.81, Fig. 2A). Decrease in toughness is moderately correlated with fibrillar stiffening (r^2^ = 0.51, Fig. 2B) and not very correlated with AGEs content (r^2^ = 0.26, Fig. 2C). This brings evidence that AGEs accumulation directly leads to reduced collagen fibril deformation (reduced plasticity and resistance to fracture). However, collagen deformation itself is not the only contributor to bone toughness, although it is an important one. Structural deficits and remodeling impairment are also key contributors to bone fragility. AGEs are detrimental because they can also affect cells responsible for remodeling and microstructure, as explained in the following section.

## Other Effects of Advance Glycation End-products Accumulation on Bone Cell Function and Geometry/ Microstructure

Excessive AGE cross-link accumulation could affect the bone cells’ ability to maintain optimal geometry and microarchitecture via unbalanced matrix resorption/formation and favors bone fragility. AGEs can alter osteoclast differentiation and reduce resorption (Valcourt *et al*., 2007; Ural *et al*., 2015), reduce the phenotypic expression of osteoblasts (Katayama *et al*., 1996), impact the osteocyte mechano-sensitivity (Hie *et al*., 2011; Tanaka *et al*., 2015; Yang *et al*., 2021), trigger cell senescence (Almeida and O’Brien, 2013; Teissier *et al*., 2022), and stimulate secretion of pro-inflammatory and catabolic factors (Takagi *et al*., 1997). AGEs also affect the collagen surface and cell-matrix interactions, impairing bone repair (Snedeker and Gautieri, 2014). Therefore, AGE accumulation with diabetes and age may impair cortical bone geometry, microstructure, and damage repair leading to a decreased resistance to fracture. More research is needed to investigate whether and how AGEs can directly impact cortical bone geometry and microstructure with age and diabetes.

## Discussion and Conclusion

We showed here that the dramatic reduction in cortical bone toughness and bone quality with age and diabetes is primarily attributable to deficits in material properties, specifically in collagen-related plasticity, and secondly to structural deficits and remodeling impairment. Non-enzymatic cross-linking plays a key role in these three aspects of bone quality (material properties, structure, and remodeling). The studies we combined in this viewpoint consistently show a direct relationship between the accumulation of AGEs and the reduction of fibrillar deformation, which is closely associated with the loss of toughness and plasticity. AGEs are also known to affect the cells responsible for bone formation, resorption, and damage repair leading to structural changes. This contribution of AGEs to toughness via remodeling is not well quantified yet. This knowledge would give evidence that AGEs affect bone quality via different mechanisms and thus are key regulators of bone quality.

Studies have reported many limitations that hinder the ability to draw further conclusions. While AGEs are commonly measured in serum, urine, and the skin, currently, no strong correlation between AGEs in bone and these measurable AGEs has been noted (Kida *et al*., 2019; Waqas *et al*., 2020). AGEs content in bones varies greatly from one study to another (Karim and Bouxsein, 2016); the content can be pentosidine (via HPLC) or bulk fluorescent AGEs (combining pentosidine, CML, CEL, crossline, and vesper lysines A, B, and C). However, it is not clear which AGE is the most important or whether pentosidine is the right target to study. Indeed, pentosidine has been the most studied AGEs even though it represents less than 1% of fluorescent AGEs in bones (Dyer *et al*., 1991); it is only weakly correlated to the total amount of fluorescent AGEs in human cortical and cancellous bone (Karim *et al*., 2013). Glucosepane can be another promising (non-fluorescent) AGEs to investigate in bones because it has been found to be abundant in the skin of human diabetic patients (Monnier *et al*., 2014). CML seems to have the potential to be an efficient (non-fluorescent) marker of AGEs (Thomas *et al*., 2018); these (non-crosslinking) AGE adducts might be associated with the loss of enzymatic cross-links and resistance to fracture (Thomas *et al*., 2018; Willett *et al*., 2022). In general, more information about the process of AGEs cross-linking (abundance, binding sites, size, and shape) at the molecular level would help understand the mechanical effects of AGEs on the molecular collagen behavior and identify the most detrimental AGEs to target. Measuring AGEs, such as pentosidine or CML, in skin, urine, or serum may be a potential method to predict bone fragility, but further investigation is needed to confirm the efficacy of the technique.

Researchers have been exploring therapeutic agents that can inhibit the formation of AGEs, block the interaction between AGEs and the RAGE, or break down existing AGEs to restore bone resistance to fracture in diabetic or aged patients (Willett *et al*., 2022). One of the most promising therapies lately involved pyridoxamine (vitamin B6) (Tanimoto *et al*., 2007; Nagai *et al*., 2012; Mascolo and Vernì, 2020). Whether or not current anti-resorptive treatments such as bisphosphonate (used to treat elderly and diabetic patients) have a beneficial or detrimental effect on AGEs accumulation is still under debate. In our combined study (Fig. 2C), we observed conflicting results; long-term bisphosphonate in healthy dogs increased AGEs, whereas bisphosphonate treatment in osteoporotic patients decreased AGEs.

In conclusion, further research is necessary to fully understand the molecular mechanisms by which AGEs affect the molecular functions of collagen in aging and diabetic bone fragility. This knowledge will help us identify whether AGEs quantification and removal have a clinical future in fracture prediction and therapies development to restore the biomechanical properties of bones. Improving our understanding of these mechanisms will be crucial for developing effective strategies for improving bone health in diabetic and aged individuals.
